# Editorial: Molecular mechanisms underlying exercise-alleviated sarcopenic obesity

**DOI:** 10.3389/fendo.2024.1498615

**Published:** 2024-10-14

**Authors:** Brisamar Estébanez, Chun-Jung Huang, María J. Cuevas

**Affiliations:** ^1^ Institute of Biomedicine (BIOMED), University of León, León, Spain; ^2^ Exercise Biochemistry Laboratory, Department of Exercise Science and Health Promotion, Florida Atlantic University, Boca Raton, FL, United States

**Keywords:** aging metabolism, body mass index - BMI, muscle mass and function, physical activity, resistance training (strength)

Sarcopenic obesity (SO), characterized by a concurrent decline in muscle mass and function alongside an increase in body fat, has emerged as a pressing health concern reaching an incidence of 11% worldwide (1). This multifaceted condition encompasses intricate metabolic, physiological, and molecular alterations that collectively elevate the risk of age-related diseases and contribute to an overall decline in well-being (2). Within this complex scenario, nutrition and physical activity have been recognized as key determinants to ameliorate the consequences of SO (3, 4). The primary objective of this Research Topic was to elucidate the pivotal role of exercise in addressing the pressing issue of SO and its intricate connection with accelerated aging.

The study by Rao et al. makes a significant contribution to the field of molecular mechanisms involved in SO and premature aging, by identifying genetic causal associations between basal metabolic rate (BMR), appendicular muscle mass (ALM), and benign prostatic hyperplasia (BPH). The observation that increases in BMR and ALM are associated with a higher risk of developing BPH suggests that these factors may be key indicators of the progression of SO and its impact on metabolic aging. Furthermore, the lack of a causal relationship between fat-free mass distribution and BPH highlights the complexity of the role of body composition in the pathogenesis of BPH. This study pioneers the use of Mendelian randomization to explore these associations, providing new insights into how SO might influence metabolic health and aging, especially in populations at risk for developing BPH. By increasing muscle mass, exercise may improve the muscle-to-fat ratio, elevate BMR, and alter the dynamics between BMR, ALM, and BPH, which may reduce the risk of conditions associated with SO and potentially prevent BPH.

The research by Xiang et al. provides important insights into SO by demonstrating that osteocalcin (OC) plays a protective role in preserving muscle mass during weight loss, especially in men with normal fasting glucose. The findings indicate that, despite a reduction in fat mass and a slight decrease in total muscle mass, OC is positively associated with skeletal muscle mass, suggesting its potential to counteract sarcopenia in the context of obesity. A high-protein, energy-restricted diet, which significantly improved metabolic indicators such as insulin resistance, also helped preserve muscle mass during weight loss, which is crucial for managing SO. Although the study controlled for the effect of exercise by requiring participants to take 10,000 steps daily, it is important to consider that physical exercise, especially resistance training, has a significant effect on muscle mass and hormonal regulation. Including a more detailed assessment of exercise type, intensity, and frequency in future research may offer a more complete view of how exercise interacts with diet and OC to combat SO and improve metabolic and muscular outcomes.

The review conducted by Liu et al. highlights the crucial role of physical exercise in the prevention and treatment of sarcopenia and its associated forms, such as type 2 diabetes-related sarcopenia and disuse muscle atrophy. Exercise, especially resistance training, is recognized as an effective intervention to slow the progression of muscle loss by stimulating muscle protein accumulation, improving muscle protein synthesis, and enhancing muscle quality, strength, balance, and endurance. It is emphasized that exercise activates the Akt/mTOR signaling system and reduces FOXO/MuRF1 expression, which favors protein metabolism and reduces muscle breakdown. However, it is noted that exercise strategies should be tailored to individual needs, taking into account the differences between types of muscle atrophy and specific metabolic conditions, such as type 2 diabetes. The importance of exercise is emphasized as a fundamental part of a comprehensive approach to improving muscle mass and quality of life in patients with sarcopenia.

The review by Wei et al. stresses the importance of physical exercise in the management of SO, highlighting that both aerobic and resistance exercise are essential to counteract the pathological aspects of this condition. Aerobic exercise, such as walking, swimming and cycling, improves cardiovascular function, reduces insulin resistance and improves muscle capacity and mass in patients with sarcopenic obesity. On the other hand, resistance exercise is particularly effective in inducing muscle hypertrophy, improving strength and promoting weight loss. Although clinical evidence specific to sarcopenic obesity is limited, a combination of both types of exercise is recommended to optimize benefits. Furthermore, exercise prescription should be individualized, with workouts that reach approximately 65-75% of the maximum heart rate and progressive approaches in resistance training.

This Research Topic summarizes current advances and knowledge regarding the role of exercise in managing SO. It integrates findings from studies that advance our understanding of the complex interactions between muscle mass, metabolic health, and obesity-related conditions. Collectively, these articles underscore the importance of an individualized exercise regimen as part of a comprehensive approach to preventing and treating SO and emphasize the need for tailored interventions that address individual metabolic and muscular needs. The findings from this Research Topic pave the way for future research aimed at optimizing exercise strategies and further elucidating the pathways by which exercise influences SO and related conditions.
